# Learning from the local: the variety and spatial pattern of vocal mimicry in songs of the invasive white-rumped shama in Taiwan

**DOI:** 10.1098/rsos.241676

**Published:** 2025-03-19

**Authors:** Bao-Sen Shieh, Shih-Hsiung Liang, Shuo-Chen Chang

**Affiliations:** ^1^ Biomedical Science and Environmental Biology, Kaohsiung Medical University, Kaohsiung, Taiwan; ^2^ Medical Research, Kaohsiung Medical University Hospital, Kaohsiung, Taiwan; ^3^ Department of Biotechnology, National Kaohsiung Normal University, Kaohsiung, Taiwan

**Keywords:** acoustic similarity hypothesis, Beau Geste hypothesis, biological invasion, *Copsychus malabaricus*, heterospecific mimicry, mimicry model selection

## Abstract

Studying the model selection, especially in multiple heterospecific mimicry, is crucial for understanding the function of vocal mimicry. Invasive songbirds with large repertoires in novel auditory environments lacking conspecifics expand their repertoires by imitating heterospecifics, offering valuable insights into model selection. This study examines vocal mimicry in the invasive *Copsychus malabaricus* (white-rumped shama), focusing on how this species selects mimicry models in Taiwan. We recorded the songs of 256 males across 26 sites in Taiwan, and their vocal mimicry of heterospecific sources was identified. Our results revealed that at least 28 animal species were mimicked, and 68% of those model species were endemic. Regarding individual mimics, 68.6% of 242 mimics imitated more than two species and 13.2% of total mimics imitated up to 4–8 species. Most mimicry types (defined by species mimicked) exhibited a significant clumped distribution, except three mimicry types. As the number of observed *C. malabaricus* at a site increased, the number of identified mimicry types increased significantly. Furthermore, as the total number of mimics of the two sites increased, the compositions of mimicry types of the two sites were more likely dissimilar. We suggest that individual differences play a crucial role in the model selection of heterospecific mimicry, and these differences may result from variations in individual learning ability or preferences, or from variations in the local auditory environment where the individuals inhabit.

## 1. Introduction

Vocal resemblance refers to the similarity in sounds observed across different organisms, whether conspecifics or heterospecifics, and is particularly widespread in songbirds [1–4]. This resemblance across species may arise because of convergent evolution driven by environmental adaptation or through learning. Vocal mimicry specifically refers to sound similarity between species resulting from learning rather than environmental convergence [5].

Birds that mimic the sounds of other species may imitate a single species. When imitating a single species, the selection of the mimicked species and the functional explanation for the mimicry are more apparent and straightforward. By contrast, for birds that mimic the sounds of multiple species, such as *Sturnus vulgaris* (European starling) [6], Menuridae (lyrebird) [7,8], *Ptilonorhynchus maculatus* (spotted bowerbird) [9] and *Mimus polyglottos* (northern mockingbird) [10], which learn their mimetic repertoire from more than 10 heterospecifics, the model selection and functional explanation are comparatively much more complex. An investigation of the pattern of model selection is essential to reveal the functional significance of vocal mimicry [3,8,9,11].

Furthermore, research on birds mimicking the sounds of multiple species, such as those mentioned above, has been conducted from the perspective of mimics in their native habitat; rarely have they been investigated from the perspective of mimicked species and mimics in new environments. Invasive birds in introduced environments offer a unique opportunity to investigate the model selection issue in vocal mimicry. Previous studies on the songs of invasive bird species have often focused on exploring and comparing the differences between the songs of introduced populations in new environments and those of native populations in their original habitats, such as *Fringilla coelebs* (chaffinch) [12,13] and *Emberiza citronella* (yellowhammer) [14,15]. The results of these studies indicate that the songs in invaded regions are indeed significantly different from those in their native habitats, and they may retain dialect forms that have been lost in their original habitats. However, there is currently no research on how these invasive bird species innovate their songs in introduced regions. In addition, the aforementioned invasive species, *F. coelebs,* and *E. citronella*, are close-ended learners with small repertoires and simple songs. Previous studies have shown that song learners with large repertoires such as *St. vulgaris* [6], lyrebirds [7,8], *Pt. maculatus* [9] and *Mi. polyglottos* [10] can expand their repertoires by imitating multiple heterospecifics. However, these studies were conducted exclusively in the species’ native ranges, with none examining their heterospecific mimicry in introduced regions. Invasive species with large repertories provide a valuable opportunity to investigate their model selection for vocal mimicry when encountering new auditory environments.

*Copsychus malabaricus* (white-rumped shama) comprises 12 subspecies native to India, Sri Lanka, southern China, Indonesia, Thailand, Malaysia and Vietnam [16]. Renowned for its singing ability, this species has been a popular pet and has been exported worldwide for a long time [17]. It has successfully invaded many areas, including Oahu and Kauai, in the Hawaiian Islands [16]. Males and females of the species have distinct appearances, with males of different subspecies exhibiting slight variations in crown colour, ranging from entirely black to having white crowns. However, all males share the characteristics of a white rump, long black tail and orange belly [16]. The most commonly observed subspecies in Taiwan is *C. malabaricus malabaricus*, distinguished by its all-black head. Female adults of this subspecies have similar coloration to males but with lighter back and belly colours and less pronounced colour contrast. According to the records of the Chinese Wild Bird Federation, the first record of *C. malabaricus* in Taiwan dates back to 1988 [18]. They are mainly found in green lands below 400 m above sea level on the main island of Taiwan. The breeding success rate of a nest (defined as producing at least one live fledgling) can reach 49% [19], higher than the 41.3% success rate observed in artificial nest boxes within the species’ native range [20]. In 2007, the Taiwan Endemic Species Research Institute initiated a removal project, and at least 872 *C*. *malabaricus* were removed from the wild of central and southern Taiwan during 2008−2011 (reviewed in [21]). Despite the cessation of the removal project, *C. malabaricus* has remained a highly successful invasive bird species in Taiwan. In this study, we focused on heterospecific mimicry in songs of *C. malabaricus*.

We surveyed and recorded songs of *C. malabaricus* across sites with varying population sizes to achieve three objectives: (i) to describe and compare the variety of model species mimicked (hereafter referred to as mimicry types) across sites; (ii) to examine the spatial patterns of mimicry types in terms of their distributions and their composition at each site; and (iii) to evaluate three leading hypotheses regarding mimicry model selection in the songs of this invasive species: acoustic similarity, passive sampling and proximity [10]. The acoustic similarity hypothesis, widely supported across various songbird lineages [10], proposes that mimics preferentially imitate heterospecific sounds that are acoustically similar to their own sounds. The passive sampling hypothesis, proposed for various species [6,9,10], suggests that mimics imitate heterospecific sounds they hear frequently. The proximity hypothesis predicts that mimics favour sounds from heterospecific models they physically encounter [6]. However, previous studies have yielded inconclusive results, with some rejecting this hypothesis based on individual interactions in *M. polyglottos* [10]. We analysed the variety and prevalence of mimicry types across sites to evaluate the acoustic similarity and passive sampling hypotheses. According to the acoustic similarity hypothesis, the most frequently mimicked species would be those acoustically similar to *C. malabaricus*. By contrast, the passive sampling hypothesis predicts the most frequently mimicked species would be those dominating the acoustic environments. Both hypotheses further predict a clumped spatial distribution of a mimicry type, arising from either the aggregation of acoustically similar conspecifics (acoustic similarity) or shared acoustic environments (passive sampling). Conversely, a random spatial distribution of a mimicry type would provide evidence against both hypotheses. For the proximity hypothesis, we examined the spatial patterns of mimicry types at the site (population) level. This hypothesis predicts that populations of geographically closer sites will exhibit greater similarity in the composition of mimicry types owing to increased opportunities for direct encounters with the same heterospecific models.

## 2. Material and methods

### 2.1. Field recording

**Figure 1.**
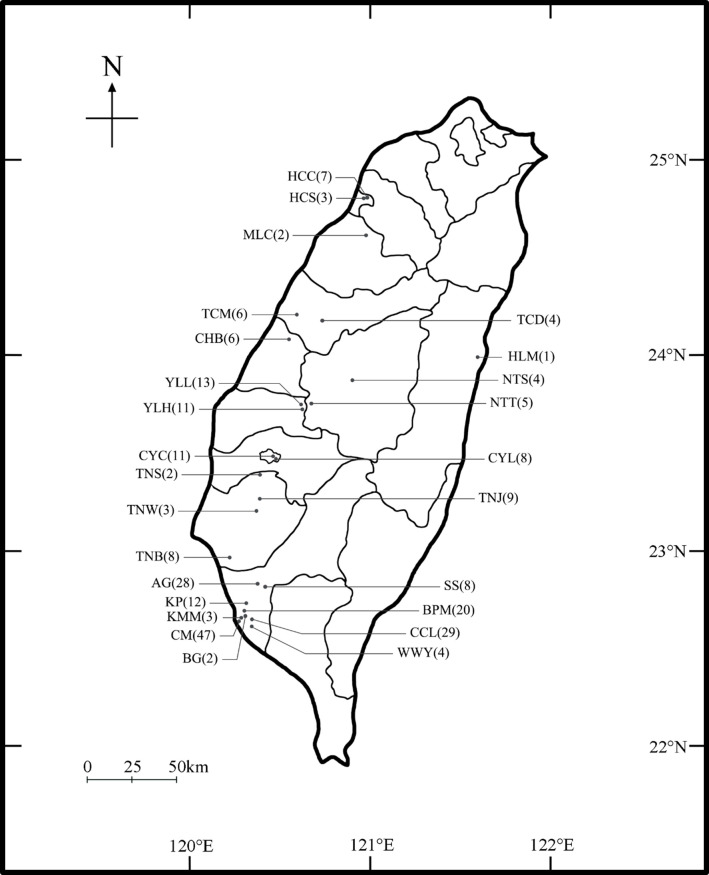
Map of sampling sites with the number of observed *C. malabaricus* in parentheses.

Before conducting field recordings, we gathered sighting records of *C. malabaricus* from three sources: (i) Taiwanese websites; (ii) the database of the Chinese Wild Bird Federation; and (iii) relevant publications [18,19,21]. In March 2022, we began recording at sites with prior *C. malabaricus* sighting records on the main island of Taiwan. We used digital recorders (Denon Portable IC Recorder DN-F20R or Marantz PMD661 connected to a Sennheiser ME67 directional microphone and Sony PCM-D100) for recording at 26 sites during the 2022 breeding season (March–July; figure 1).

Males of *C. malabaricus* exhibit distinct appearances and demonstrate stronger territorial defence behaviour compared with females [22,23]. Males remain in their territories year-round, whereas females are present only during breeding. Additionally, males produce complex, long songs in territorial advertisement and respond aggressively to song playbacks, whereas females do not [22,23]. We focused on birds suspected to be *C. malabaricus* based on their song. We then identified birds, confirmed species and sex through physical characteristics and territorial defence behaviour, and then recorded their songs until the bird flew away, or at least 20 song repetitions were documented. If the bird stopped singing, we played back its previously recorded song to stimulate further singing, collecting more song samples. For recording at a newly established site where males were scarce, we played a fixed song (containing only the most common mimicry type, TSB) to detect the presence of *C. malabaricus* quickly. Once a male responded with songs, we stopped the playback of the fixed song. Although we did not mark individuals, the strong territorial singing behaviour of *C. malabaricus* and our focal individual recording method allowed us to differentiate individuals by marking recording points on a map. Individuals were distinguished based on location. When two neighbouring males sang nearby, we used two digital recorders to record their songs separately and marked them at two nearby locations on the map (indicating the minimum distance between the two males). We searched for the species along a fixed route at each site to record their songs, conducting the search only once during the 2022 breeding season without revisiting the same locations. Males recorded from different locations within a site during this sampling period were treated as separate individuals.

### 2.2. Mimicry type and data analysis

Songs of 256 males were recorded in the 26 sites in Taiwan (figure 1). Vocal mimicry of heterospecific sources was identified by ears and by comparing the spectrograms of the mimetic song with sounds in our reference recordings (http://www.taisong.org/). We named the mimicry type according to the mimicked species (see the electronic supplementary material; table S1 for the mimicked species and mimicry type, and table S2 for spectrograms of sound examples for each mimicry type), and the composition of mimicry types in each mimic’s songs was then obtained and used for the following analysis.

First, for the spatial distribution of a mimicry type, the relative locations of mimics (242 out of 256 males) were noted on the Google map, and the latitude and longitude of each mimic location were used to calculate the distances among them. Each mimicry type used by at least two mimics was grouped into its own dataset. These datasets were analysed to determine whether mimics using the same mimicry type were clumped or randomly distributed, using nearest neighbour distance analysis [24,25]. We performed the analysis for each mimicry type and calculated the aggregation index (*R*). The null hypothesis of random distribution was tested at a significance level of 0.05. A distribution was considered clumped when |*z*| > 1.96 and *R* < 1 and uniform when |*z*| > 1.96 and *R* > 1.

Second, we compared mimicry composition, geographical distance and the total number of mimics across 26 sites to construct three 26 × 26 matrices. Mimicry composition at each site was determined by counting the number of mimics for each of the 28 mimicry types (electronic supplementary material, table S1). Pairwise comparisons of mimicry compositions across the 26 sites were used to calculate Euclidean distances through hierarchical clustering, resulting in a mimicry composition distance dissimilarity matrix (hereafter referred to as the composition dissimilarity matrix). The geographical distance matrix was based on the shortest distance between singing individuals from different sites. By contrast, the total mimics matrix reflected the sum of mimics recorded from the two paired sites. The correlation between the composition dissimilarity matrix and the geographical distance matrix and the correlation between the composition dissimilarity matrix and the total mimics matrix were investigated separately using the Mantel test (2000 permutations, Spearman correlation), with two-tailed empirical probabilities reported.

Furthermore, we performed Spearman’s rank correlation analyses on the 26 sites to examine the correlation between the number of observed male shamas and the number of mimicry types at each site. Additionally, for the 28 mimicry types, we examined the correlation between the aggregation index of each mimicry type and the number of mimics using that type. All statistical analyses were performed using JMP Pro 17.0, with a significance level set at 0.05.

#### 2.2.1. Ethics statement

This study was conducted entirely in the field and did not involve the handling or collection of animals. It was reviewed, approved and funded by the Ministry of Science and Technology, Taiwan, ROC (grant no. MOST 110-2311-B-037-002-MY3). All research activities complied with the legal requirements of Taiwan.

## 3. Results

### 3.1. The variety of heterospecific mimicry

Most (99.6%) male shamas were observed in the field of western Taiwan, and only one in eastern Taiwan (figure 1). Among 26 sites observed with male shamas, 19 sites were found to have the percentage of mimics as 100%, that is, all observed males in the population with heterospecific mimicry in their song (table 1). Approximately 95% of the observed males (242 out of 256) were found to have heterospecific mimicry in their songs. Regarding individual mimics, 68.6% of total mimics (242 mimics) imitated more than two heterospecific species and 13.2% of total mimics imitated up to 4−8 species. As to all the 242 mimics, we identified 28 heterospecific models mimicked, including 26 avian models, one mammal model and one insect model (table 2; see the electronic supplementary material, table S2 for the links of audio files for song examples of each mimicry type and their corresponding spectrograms). Among those animal models, 68% (19 out of 28) of the model species are endemic to Taiwan. Furthermore, the proportion of mimics associated with each model species—calculated as the number of mimics for a model species relative to the total number of mimics—showed that the top seven species with the highest proportions were all endemic. The two species mimicked by more than 50% of the total mimics were *Pomatorhinus musicus* (Taiwan scimitar-babbler) and *Pycnonotus sinensis* (light-vented bulbul) (table 2).

**Table 1.** The total number of observed *C. malabaricus* and mimics found in the field in Taiwan in 2022.

site	no. of observed shamas	no. of mimics	proportion of mimics	no. of mimics				
				1 model species	2 model species	3 model species	4−8 model species	
CM	47	46	0.98	14	12	14		6
CCL	29	27	0.93	4	14	4		5
AG	28	24	0.86	13	8	1		2
BPM	20	19	0.95	2	9	1		7
YLL	13	13	1.00	4	6	3		0
KP	12	8	0.67	2	5	1		0
CYC	11	11	1.00	5	2	2		2
YLH	11	10	0.91	3	6	1		0
TNJ	9	9	1.00	3	3	3		0
SS	8	7	0.88	2	4	1		0
TNB	8	8	1.00	0	4	3		1
CYL	8	8	1.00	7	1	0		0
HCC	7	7	1.00	2	2	2		1
TCM	6	6	1.00	2	3	0		1
CHB	6	6	1.00	1	0	2		3
NTT	5	5	1.00	3	2	0		0
WWY	4	4	1.00	2	1	0		1
TCD	4	4	1.00	0	3	1		0
NTS	4	4	1.00	1	1	1		1
KMM	3	3	1.00	0	3	0		0
TNW	3	3	1.00	1	0	1		1
HCS	3	3	1.00	2	0	1		0
BG	2	2	1.00	2	0	0		0
TNS	2	2	1.00	0	0	1		1
MLC	2	2	1.00	1	1	0		0
HLM	1	1	1.00	0	0	1		0
total	256	242	0.95	76	90	44		32

**Table 2.** A list of model species or taxa mimicked in songs of 242 mimics in 26 sites in Taiwan. Model species are ordered from the most frequently to the least frequently sung by the mimics. Mimicry types were defined by the model species or taxa mimicked.

model species/taxon	mimicry type	status	no. of mimics	proportion of mimics	no. of sites	proportion of sites
*Pomatorhinus ruficollis musicus*	TSB	endemic	220	0.909	26	1.000
*Pycnonotus sinensis formosae*	WBU	endemic	148	0.612	25	0.962
*Stachyris ruficeps praecognitum*	RB	endemic	25	0.103	13	0.500
*Hypothymis azurea oberholseri*	BCM	endemic	24	0.099	11	0.423
*Alcippe morrisonia morrisonia*	GCF	endemic	17	0.070	5	0.192
*Bambusicola thoracicus sonorivox*	BPA	endemic	14	0.058	7	0.269
*Dicrurus macrocercus harterti*	BD	endemic	11	0.045	9	0.346
*Prinia flaviventris*	YB		9	0.037	6	0.231
*Pomatorhinus erythrogenys erythrocnemis*	BSB	endemic	8	0.033	6	0.231
*Zosterops japonicus*	JWE		8	0.033	4	0.154
*Megalaima nuchalis*	TB	endemic	7	0.029	6	0.231
*Garrulax taewanus*	HM	endemic	7	0.029	2	0.077
*Dendrocitta formosae formosae*	GT	endemic	6	0.025	5	0.192
*Abroscopus albogularis*	WFW		4	0.017	4	0.154
*Oriolus chinensis*	HL		4	0.017	4	0.154
*Spilornis cheela hoya*	CE	endemic	4	0.017	3	0.115
*Hypsipetes leucocephalus nigerrimus*	BB	endemic	3	0.012	3	0.115
*Schoeniparus brunneus*	GF		2	0.008	2	0.077
*Passer montanus*	TS		2	0.008	2	0.077
*Gallus gallus domesticus*	Hen	domestic	2	0.008	2	0.077
*Alcedo atthis*	KF		2	0.008	1	0.038
*Pericrocotus flammeus*	PF		1	0.004	1	0.038
*Copsychus saularis*	CC	invasive	1	0.004	1	0.038
Cacatuidae	CAC	invasive	1	0.004	1	0.038
*Heterophasia auricularis*	WES	endemic	1	0.004	1	0.038
*Pycnonotus taivanus*	SBU	endemic	1	0.004	1	0.038
*Callosciurus erythraeus thaiwanensis*	SQ	endemic	1	0.004	1	0.038
Gryllidae	CRI		1	0.004	1	0.038

### 3.2. The spatial pattern of mimicry type

**Figure 2.**
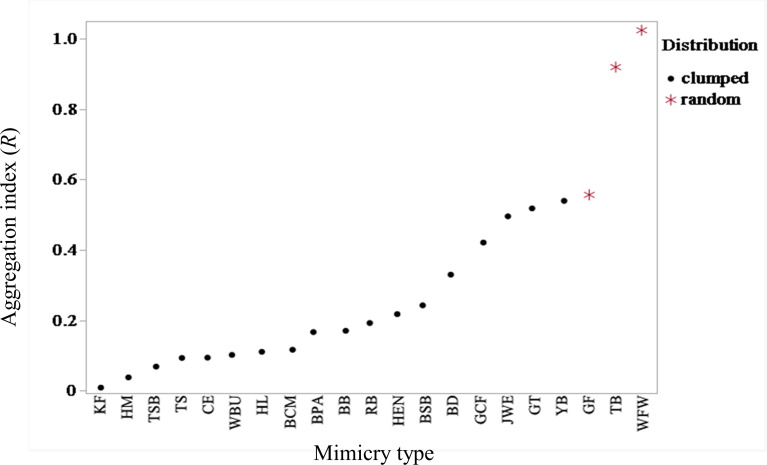
The aggregation index (*R*) for each mimicry type, which is defined by the model species mimicked (table 2). The distribution of each mimicry type was tested against the null hypothesis of random distribution at a significance level of 0.05. Distributions were categorized as clumped when |*z*| > 1.96 and *R* < 1, and as uniform when |*z*| > 1.96 and *R* > 1.

We named the mimicry type according to the model species mimicked (see table 2 for scientific names of the model species or taxa mimicked and their corresponding mimicry types). The aggregation index (*R*) for the 21 mimicry types with more than one mimic ranged from 0.008 for mimicry type KF, corresponding to the model species *Alcedo atthis*, to 1.025 for mimicry type WFW, corresponding to the model species *Abroscopus albogularis* (figure 2). The aggregation index of each mimicry type had no significant relationship to its number of mimics (Spearman correlation coefficient = −0.035, *p* = 0.88). In tests of significance for deviation from randomness, most mimicry types (18 out of 21) exhibited a significantly clumped distribution (*p* < 0.05, *R* < 1). By contrast, only three mimicry types—WFW (model species *Ab. albogularis*), TB (model species *Megalaima nuchalis*) and GF (model species *Schoeniparus brunneus*)—showed a random distribution (*p* > 0.05).

**Figure 3.**
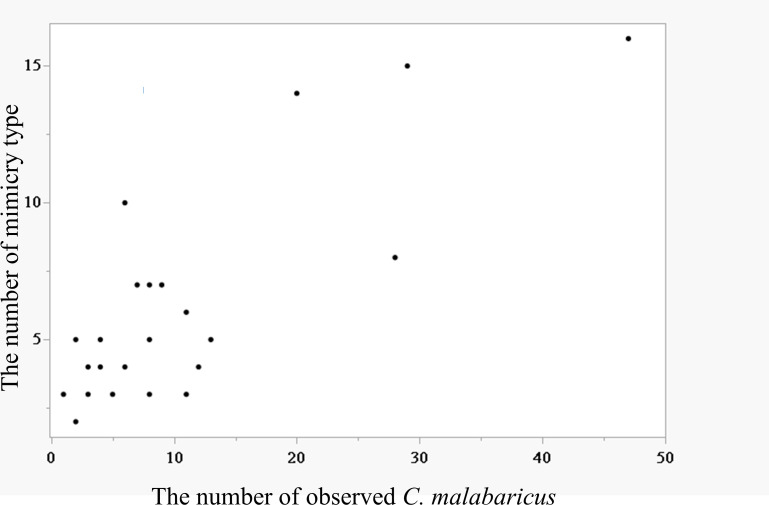
The relationship between the number of mimicry types and the number of observed *C. malabaricus* at each site. The correlation was significant (Spearman correlation coefficient = 0.67, *p* < 0.001).

**Figure 4.**
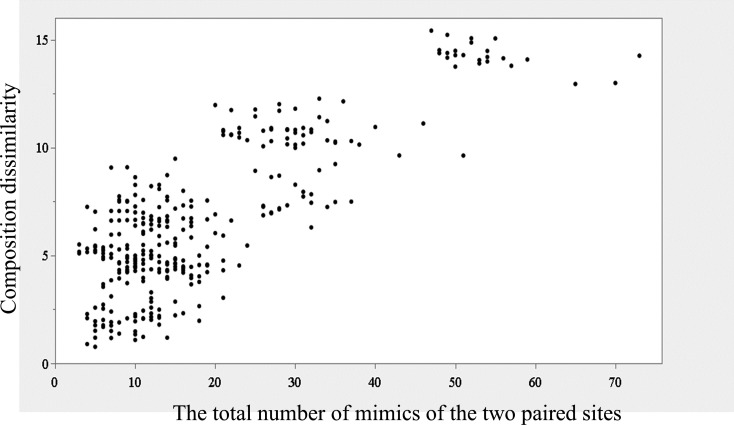
The relationship between the composition dissimilarity and the total number of mimics of the two paired sites. Using pairwise comparisons across 26 sites, we generated two 26 × 26 matrices: one for composition dissimilarity and the other for the total mimics. The composition dissimilarity matrix was derived by calculating Euclidean distances using hierarchical clustering on pairwise mimicry composition comparisons. The total mimics matrix was created by summing the number of mimics recorded at the two paired sites. A Mantel test revealed a significant correlation between the two matrices (Spearman correlation coefficient = 0.66; number of permutations = 2000; empirical *p* < 0.001).

As the number of observed *C. malabaricus* at a site increased, the number of identified mimicry types increased significantly (Spearman correlation = 0.67, *p* < 0.001; figure 3). Regarding the mimicry compositions and geographical distances across 26 sites, we found that the composition dissimilarity matrix was not significantly correlated with the geographical distance matrix (Mantel test, Spearman correlation coefficient = −0.0231, permutation number = 2000, empirical prob. = 0.674). However, it was significantly positively correlated with the total mimics matrix (Mantel test, Spearman correlation coefficient = 0.66, permutation number = 2000, empirical prob. < 0.001). This demonstrated that the mimicry composition of a site was more likely to be dissimilar to that of another site as the total number of mimics—calculated as the sum of mimics from the two paired sites—increased (figure 4).

## 4. Discussion

Vocal mimicry is common in birds, but previous research has shown that model selection for species capable of imitating a wide range of heterospecific sounds is complex and crucial for understanding the function of vocal mimicry [3,8,9,11]. When a bird species invades a new environment, the selection of models for vocal mimicry offers an excellent opportunity for study. This research explored how *C. malabaricus*, a species renowned for its singing abilities and invasive in Taiwan for over 30 years, selected its mimicry sources in a new environment. Our results revealed that, at the individual level, almost all shamas (95%) engaged in heterospecific mimicry, with most mimicking more than two species and 13.2% of mimics imitating as many as 4−8 different species. Collectively, the shams mimicked up to 28 different animal species, with the majority (68%) being Taiwan endemic species. Individuals that mimicked the same species tended to be geographically clustered, although some exhibited random distribution. However, the degree of this aggregation was not related to the number of mimics. At the population level across 26 sites, there was a significant positive correlation between the number of species mimicked and the number of observed *C. malabaricus* at each site. Additionally, the difference in composition between sites was not related to geographical distance but was associated with the total number of mimics in the two sites. Our findings, both at the individual and population levels, confirmed that the invasive *C. malabaricus* in Taiwan mimicked multiple heterospecific species, which are more likely Taiwan endemic, and the number and composition of species mimicked were more closely related to the number of individuals performing the mimicry than to geographical distance.

Previous research on the proximate factors influencing model selection in vocal mimicry proposed five hypotheses [10]. This study aimed to evaluate three of these hypotheses: the acoustic similarity hypothesis, the passive sampling hypothesis and the proximity hypothesis. Our findings supported the acoustic similarity hypothesis and the passive sampling hypothesis while rejecting the proximity hypothesis.

The acoustic similarity hypothesis was first supported by Dowsett-Lemaire [26] and later in various studies (e.g. [6,10,27,28]). This hypothesis proposes that mimics preferentially imitate heterospecific sounds that are acoustically similar to their own. After examining different lineages of birds with vocal mimicry, Gammon [10] suggested that the acoustic similarity hypothesis could serve as a general rule to explain the evolution of vocal mimicry. In this study, we provide two lines of evidence supporting this hypothesis. First, 90% of *C. malabaricus* individuals imitated the sounds of *Po. musicus* (mimicry type TSB). Shieh [29] reported that the song structure of *Po. musicus* more closely resembles that of *C. malabaricus* than those of the other two endemic babbler species in Taiwan, *Alcippe morrisonia* and *Garrulax taewanus*. The second finding is that the species being mimicked include nearly all lowland resident species in Taiwan, except for doves. Although native dove species were commonly present at the sampling sites, their cooing sounds, which have a peak frequency range of less than 0.8 kHz [30], do not resemble *C. malabaricus*’s peak frequency range of over 1 kHz and thus were not mimicked. Another hypothesis that cannot be ruled out is the passive sampling hypothesis. This hypothesis suggests that mimics preferentially imitate heterospecific sounds they hear frequently [6,9,10]. Evidence supporting this hypothesis includes the observation that the second most frequently mimicked species was *Py. sinensis*. Mimicking individuals were observed at all 25 sampling sites in the lowland greenlands of western Taiwan, where *Py. sinensis* was a dominant songbird, singing year-round for territorial advertisement [31]. This species is the most common and frequently heard resident species at these sites, suggesting that frequent auditory exposure increases the likelihood of it being mimicked. The proximity hypothesis suggests that mimics favour sounds from heterospecific models they physically encounter. We found no significant correlation between the mimicry composition dissimilarity of the paired sites and the geographical distance of those paired sites across 26 sites (populations), providing no evidence to support the proximity hypothesis at the population level.

The other two hypotheses related to proximate factors influencing model selection in vocal mimicry, proposed in previous studies but not evaluated in the present study, are the alarm hypothesis and the interspecific aggression hypothesis [10]. The alarm hypothesis was excluded from consideration because the observed vocal mimicry occurred in a territorial rather than an alarm context. The interspecific aggression hypothesis posits that mimics preferentially imitate heterospecific models involved in aggressive encounters with them [9,10]. While this hypothesis was not explicitly evaluated in the present study, our field observations and previous research provided evidence to reject it for our study species. First, during field observations and recording, we witnessed aggressive chasing between individuals of *C. malabaricus* but not between *C. malabaricus* and other species. Furthermore, most individuals mimicked more than one heterospecific sound in their songs, suggesting that mimicking several heterospecific sounds might be ineffective for territorial aggression towards a single heterospecific species (e.g. [1,32]) owing to insufficient resemblance. Shieh [29] found that broadcasting the songs of *C. malabaricus* to three local babbler species elicited far fewer responses than broadcasting the songs of those three local babbler species. Therefore, the interspecific aggression hypothesis might be more applicable to the mimicry of a single heterospecific species, leading us to reject it for our study species.

Although our findings on the two most preferentially mimicked species, *Po. musicus* and *Py. sinensis*, provided evidence supporting the acoustic similarity and passive sampling hypotheses, respectively, the three mimicry types—WFW (*Ab. albogularis*), TB (*Me. nuchalis*) and GF (*Sc. brunneus*)—exhibited random distributions inconsistent with these two hypotheses. This inconsistency arises because both hypotheses predict a clumped distribution of mimicry types, driven either by the aggregation of acoustically similar conspecifics (acoustic similarity hypothesis) or by shared acoustic environments (passive sampling hypothesis). These three mimicry types were rarely mimicked, occurring in less than 3% of the total mimics (table 2). Our findings suggest that the selection of model species in multiple heterospecific mimicry may be shaped not only by multiple non-exclusive hypotheses, as proposed in previous studies (e.g. [28]), but also by an additional key factor. We propose that this factor is individual differences, which probably play a crucial role in expanding the repertoire of heterospecific models through the selection of rare mimicry types. The following findings support this suggestion. We observed that the greater the number of observed shamas, the more species were mimicked. Moreover, the dissimilarity in mimicked species composition between paired sites was positively correlated with the total number of mimics at these two sites, indicating that as the number of individuals increases, so does individual variability, leading to greater dissimilarity in the composition of mimicked species between sites. This variability in mimicked species caused by individual differences could stem from differences in learning ability or preferences, or from variations in the local auditory environment where the individuals inhabit.

Regarding the functional explanation of vocal mimicry in songs, previous research has rarely explored vocal mimicry as part of the sexual display [33]. Although it has been proposed that vocal mimicry enhances song complexity to attract females [1,4], some studies have suggested that heterospecific mimicry in songs serves no significant function in female choice and may simply result from mistakes made during song learning, leading to the learning mistakes hypothesis [3,4,34]. Since the mistakes were made through learning, as suggested by the hypothesis, we can expect that a population with more individuals exhibiting differences in song learning preferences and abilities will have more varied ‘mistakes’. Thus, our findings regarding the crucial role of individual variability in mimicry compositions of populations are consistent with the individual song learning aspect of the learning mistakes hypothesis, although the context differs. Our study focused on territorial contexts in intrasexual communication, whereas the original context for the learning mistakes hypothesis pertains to mate attraction in intersexual communication.

Furthermore, we suggest that when *C. malabaricus* enters a new environment, it learns territorial songs that increase its repertoire size to create the illusion of numerous conspecific competitors in its territory, as suggested by the Beau Geste hypothesis [35]. However, in the absence of nearby conspecifics in the newly invaded environment, the bird selects sounds with frequency ranges most similar to its own or the most commonly heard species in the environment to incorporate into its songs. This selection reflects individual differences, which may arise from the individual’s acoustic environment or from individual learning preferences, leading to a random distribution of mimicry types in the early invasion stage. Then, as the number of individuals in a site increases, individuals of the same mimicry type clustered simply as the result of individuals clustered in the same site. Furthermore, as the number of individuals in a site increases, more species are being mimicked, and the composition of mimicry types becomes more diverse, as our results have shown. Whether *C. malabaricus* acts like open-ended song learners who modify their song repertoires after their first year of life and whether it has greater opportunities to learn new heterospecific songs from its auditory environment requires further investigation into yearly changes in individual songs.

We suggest that *C. malabaricus*’s multiple heterospecific mimicry in territorial songs probably results from differences in individual learning environments and abilities in model selection when adapting to new environments. As inferred from the Beau Geste hypothesis, multiple heterospecific mimicry in our study species might be used to increase repertoire size for conspecific competition. However, this still requires further investigation, including whether vocal mimicry within the same territory changes over time and playback experiments to determine whether mimicry enhances territorial defence compared with non-mimicry songs.

## Data Availability

All data are provided in the manuscript and electronic supplementary material [36].
